# Self-Affinity, Self-Similarity and Disturbance of Soil Seed Banks by Tillage

**DOI:** 10.3390/plants2030455

**Published:** 2013-07-05

**Authors:** Luís S. Dias

**Affiliations:** Department of Biology, School of Sciences and Technology, University of Évora, Ap. 94, Évora 7002-554, Portugal; E-Mail: lsdias@uevora.pt; Tel.: +351-266-760-881; Fax: +351-266-760-914

**Keywords:** disturbance, fractal, seed shape, seed size, self-affinity, self-similarity, soil seed banks, tillage

## Abstract

Soil seed banks were sampled in undisturbed soil and after soil had been disturbed by tillage (tine, harrow or plough). Seeds were sorted by size and shape, and counted. Size-number distributions were fitted by power law equations that allowed the identification of self-similarity and self-affinity. Self-affinity and thus non-random size-number distribution prevailed in undisturbed soil. Self-similarity and thus randomness of size-number distribution prevailed after tillage regardless of the intensity of disturbance imposed by cultivation. The values of fractal dimensions before and after tillage were low, suggesting that short-term, short-range factors govern size-number distribution of soil seed banks.

## 1. Introduction

Plants and plant communities dynamics, strategies, processes, changes and their relationships with functional and adaptative traits can be envisioned and studied at various scales, either in space, time or both [1,2]. However, with relatively few exceptions, understanding plants and plant communities cannot be seriously attempted if seeds (in a broad, non-morphological sense, thus including fruits like cypselas or caryopses) in soil are not taken into account because soil seed banks represent in any given moment the potential population of plants [3].

Seed size is long known to be a highly heritable trait and with relatively few exceptions seed size shows very low levels of within species variability [3,4,5]. It has also long been suggested that, as a general rule, the larger the amount of reserves, and thus the larger the seed, the more advanced the stage of succession that can be occupied by a given species [6]. The ecological and functional relevance of seed size and seed shape were extensively reviewed a few years ago [7] and evidence of seed size and shape as an ecological and functional correlate have been provided among others in relation to light requirements for germination [8,9], longevity, dormancy and persistence in soil [10,11,12,13], growth form [14,15,16], ability to withstand disturbances [17,18], ability to resist removal from soil surface by water erosion [19], seedling establishment and performance [20,21,22] and plant distribution and abundance [23,24,25].

Soil seed banks are also the result of an intricate number of interactions between plants and short and long-term environmental conditions and changes [26,27], representing a “memory” of selective pressures and of plant communities responses in a timeline from, a more or less, ancient past to recent conditions [28,29]. Therefore, size-number distribution of seeds is a reflection of past events and should be sensitive and reflect disturbances as they occur.

Size-number distribution of seeds can be described either by power law or by Weibull equations [30]. However, the Weibull function [31] can reduce to the power law [32], but without the ability of the former to easily accommodate size-number distributions in which the relationship between size and number depends on and varies with size itself. Therefore, the power law was adopted to describe size-number distributions of seeds in soil seed banks.

Seed size varies across a wide range of values, which can attain for individual plant communities as much as six orders of magnitude [14,33]. Nevertheless, seed size does not extend indefinitely, especially not in the lower end of size range and therefore, strictly speaking, seed-size distribution cannot be viewed as a fractal distribution—a distribution that at any given portion is a reduced-scale but similar representation of the whole, which extends indefinitely [34]. However, the power law distribution is equivalent to a fractal distribution [32], thus allowing the use of a number of its properties and features, namely the statistical self-similarity of fractal distributions and the consequent randomness of size-number distribution of seeds [34,35]. Still borrowing from fractal geometry concepts, whenever the fractal dimension is not constant across the whole range of seeds size, multifractals and statistical self-affinity would be in place [36,37] and consequently, non randomness of size-number distribution of seeds would occur [38]. In addition, the magnitude of the fractal dimension can be informative of the scale at which governing effects are acting on size-number distribution of seeds, with high values of the fractal dimension related to short-range variation, and low values of the fractal dimension related to large-range variation [38].

Our hypothesis is that size-number distribution of seeds reflects past events including disturbances that can be detected by fitting power law equations and adopting self-similar and self-affine concepts of fractal geometry, including the magnitude of fractal dimension. Therefore, we investigated seed banks in soils not disturbed by Man for several years and evaluated the effect of soil disturbances on the type and magnitude of fractal dimensions in size-number distributions. Soil tillage was chosen as disturbance because it minimizes or completely avoids the risk of seed destruction and because it provides an easy and fast way to impose disturbances of increasing intensity. In this study we used three types of tillage—tine, harrow and plough—known to represent a series of increasing disturbance of soil structure and properties.

## 2. Results and Discussion

### 2.1. General Characterization

Overall, a total of 60,643 seeds were counted: 49% in the nine cores sampled before tillage down to 20 cm depth, 41% and 10% in the six cores sampled after tillage down to 20 cm depth (after plough and tine) and in the three cores sampled after tillage down to 10 cm depth (after harrow), respectively. Either before or after tillage, non-spherical seeds were more abundant than spherical seeds, their percentage ranging between 53% (in nine cores taken before tillage) and 62% (in three cores taken after harrowing). Given the adequacy of mesh side to estimate seed volume [30,39] seed size was calculated and found to extend almost always across three orders of magnitude, and only in one sample across four orders of magnitude.

Twenty species belonging to 10 families were identified (Table 1). Therophytes were by far the most represented, but no family was clearly dominant. According to [40] the most part of the species could be viewed as important weeds.

**Table 1:** Species, families, biological type, and importance as weed according to [40], of seeds in the soil seed bank.

Species	Family	Biological type	Importance as weed
*Amaranthus albus* L.	Amaranthaceae	Therophyte	2
*Amaranthus retroflexus* L.	Amaranthaceae	Therophyte	3
*Chenopodium album* L.	Amaranthaceae	Therophyte	3
*Senecio vulgaris* L.	Asteraceae	Therophyte	3
*Diplotaxis catholica* (L.) DC.	Brassicaceae	Therophyte	3
*Raphanus raphanistrum* L.	Brassicaceae	Therophyte	3
*Rapistrum rugosum* (L.) All.	Brassicaceae	Therophyte	2
*Sisymbrium irio* L.	Brassicaceae	Therophyte	−
*Cerastium glomeratum* Thuill.	Caryophyllaceae	Therophyte	2
*Silene gallica* L.	Caryophyllaceae	Therophyte	2
*Spergularia purpurea* (Pers.) G. Don	Caryophyllaceae	Therophyte	2
*Stellaria media* (L.) Vill.	Caryophyllaceae	Therophyte	3
*Trifolium glomeratum* L.	Fabaceae	Therophyte	−
*Juncus bufonius* L.	Juncaceae	Therophyte	1
*Plantago coronopus* L.	Plantaginaceae	Therophyte or Hemicryptophyte	2
*Cynodon dactylon* (L.) Pers.	Poaceae	Hemicryptophyte	3
*Paspalum dilatatum* Poir.	Poaceae	Hemicryptophyte	−
*Poa annua* L.	Poaceae	Therophyte or Hemicryptophyte	3
*Rumex acetosella* L.	Polygonaceae	Hemicryptophyte	1
*Reseda luteola* L.	Resedaceae	Hemicryptophyte	2

Importance as weed: 1, of minor importance; 2, important in a few situations, although it may be widespread as a minor weed; 3, important competitive weed occurring in many crops and situations [40].

No seed was found in any of the 10 random samples of the mineral fraction of the 0.297 mm or lesser mesh side and of materials not retained by the 0.149 mm mesh side. Therefore, it is highly unlikely that seed losses occurred as a result of reducing the amount of material to be screened under stereomicroscope or of not using sieves with mesh sides smaller than 0.149 mm.

### 2.2. Seed-Size Distributions

Fitting the reparameterized power function of Equation (4) to the 477 samples (396 samples 2.5-cm depth, 36 pooled samples 10-cm depth, 45 pooled samples 20-cm depth) was always possible. The adjusted coefficient of determination (*R*^2^_adj_) ranged between 0.839 and >0.999 with a mean value (±SE) of 0.977 ± 0.001. All equations met the conditions for acceptance at the first or after the second attempt. In 82% of the cases, fitted equations required only one term, 16% two terms, 2% three terms, and no equation needed the four terms of the full candidate model. Size-number distribution of seeds showed self-similarity in 44% of samples, self-affinity in 56% of samples. Whenever *D*’ was not constant across all values of seed size, meaning that self-affinity rather than self-similarity was present, values of *D*’ of Equation (6) increased monotonically with seed size in 89% of samples, the most part of them involving non-spherical seeds.

**Figure 1:**
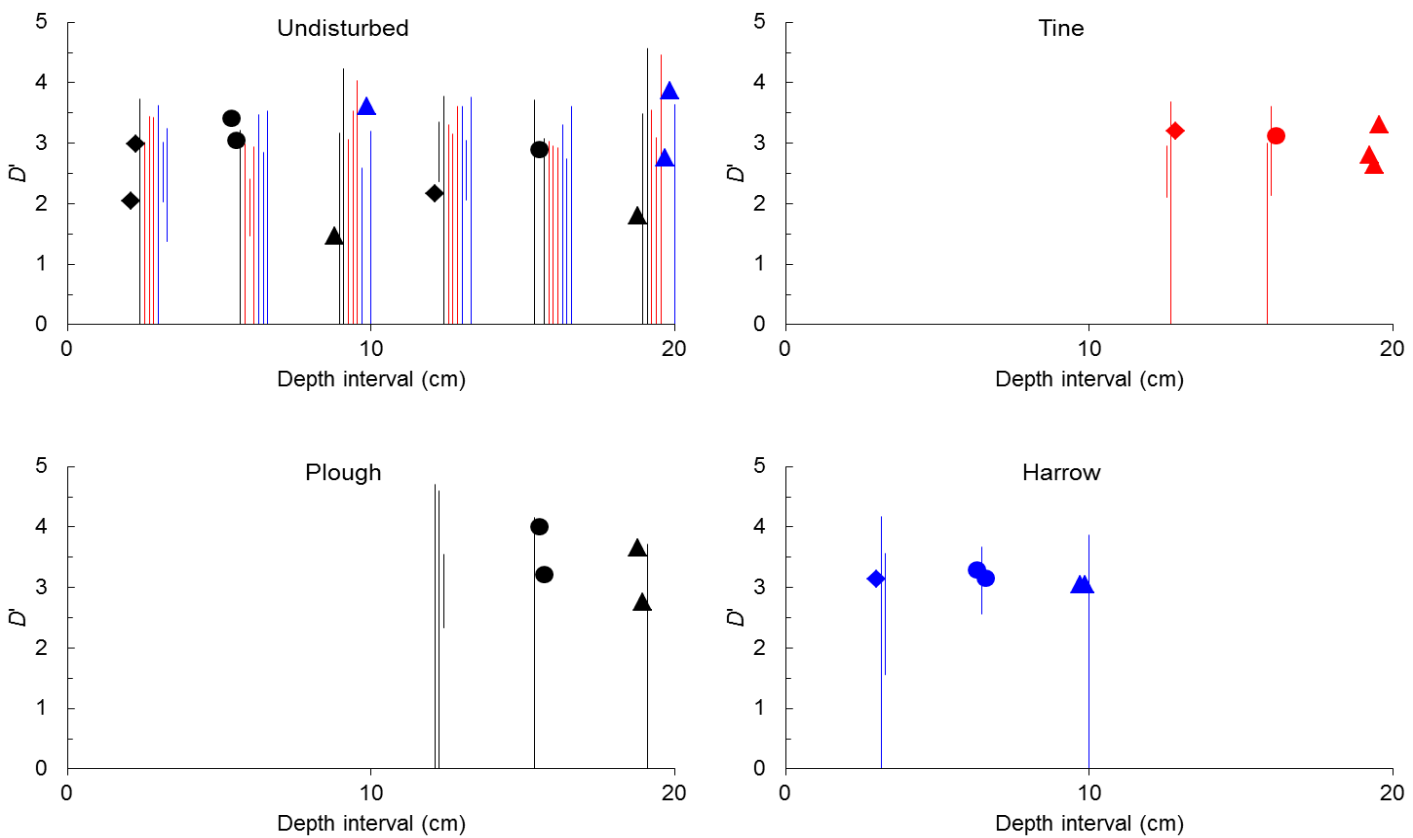
Value (points) or range (bars) of size-number distributions of seeds expressed by *D*’ for 0–10 cm or 0–20 cm soil depth before tillage (undisturbed) and after tine, harrow or plough.

However, more important than the relatively small predominance of samples in which seed-size distribution implies self-affinity is the partition of self-similarity and self-affinity between undisturbed (non-tilled) soil and tilled soil. Because harrowing was done only down to a 10 cm depth while tine and plough could be done down to 20 cm depth (see Experimental Section below) size-number distributions before tillage were modeled for 0–10 cm and 0–20 cm, with the results before and after tillage summarized in Figure 1.

Considering samples of undisturbed soil down to 10 cm depth, single values of *D*’ and thus self-similarity were found in 22% of samples regardless of the shape of seeds. However, almost all samples (83%) with self-similarity were located in the plot in which plough was to be done, simultaneously the plot located higher in the field. Considering samples of undisturbed soil down to 20 cm depth, self-similarity was found in 11% of samples of total and spherical seeds and in 33% of samples of non-spherical seeds, again with the majority of samples with self-similarity (60%) from the plot in which plough was to be done.

In general, tillage clearly increased the frequency of self-similarity. After tine, self-similarity was found in 33% of total and spherical seeds and in 100% of non-spherical seeds, while before tine, self-similarity was found in 33% or less of samples and was completely absent from the plot where tine was to be done. After harrow, self-similarity was found in 33% of total seeds and in 66% of spherical and non-spherical seeds while before harrow self-similarity was found in 22% of samples and almost completely absent from the plot where harrow was to be done. Finally, after plough self-similarity was found in 66% of spherical and non-spherical seeds while before plough self-similarity was found in 33% or less of samples and again in 33% of samples from the plot where plough was to be done.

In black, samples from the plot assigned to plough; in red, from the plot assigned to tine; in blue, from the plot assigned to harrow. In all panels (and in each depth interval of undisturbed soil) the first block of *D*’ values, including diamonds is for all seeds (spherical plus non-spherical); the second, including circles is for spherical seeds; the third, including triangles is for non-spherical seeds. Points indicate self-similarity, bars indicate self-affinity.

As for the value of *D*’ expressed as the larger value in samples with self-affinity of seed-size distribution (those with an interval of values of *D*’), no significant differences were found among total, spherical and non-spherical seeds in undisturbed soil in the depth interval 0–10 cm (*p* = 0.668, pooled mean of *D*’ 3.037 ± 0.138) and in the depth interval 0–20 cm (*p* = 0.308, pooled mean of *D*’ 3.235 ± 0.128). In addition, no significant differences were found between all samples in the depth interval 0–10 cm and 0–20 cm (*p* = 0.297).

Similarly, no significant differences were found among *D*’ of total, spherical and non-spherical seeds after tine (*p* = 0.880, pooled mean of *D*’ 2.891 ± 0.177), after harrow (*p* = 0.866, pooled mean of *D*’ 3.143 ± 0.213) and after plough (*p* = 0.772, pooled mean of *D*’ 3.684 ± 0.267).

Analyzing total, spherical and non-spherical seeds again no significant differences were found between *D*’-values before and after tine (*p* = 0.168), harrow (*p* = 0.698) and plough (*p* = 0.104).

Altogether these results suggest that soil disturbance by tillage considerably alters size-number distributions of seeds in soil shifting the distribution from predominantly self-affine in undisturbed soils to predominantly self-similar in tilled soils. On the contrary, the larger *D*’ value either from self-similar or from self-affine distributions appears to be insensitive to soil disturbance.

Probing deeper size-number distributions of seeds in soil before and after tillage involved fitting Equation (4) to all seeds (spherical plus non-spherical), and separately to spherical and non-spherical seeds at 2.5 cm soil depth intervals down to 10 cm (after harrow only) or to 20 cm (undisturbed and after plough and tine).

**Figure 2:**
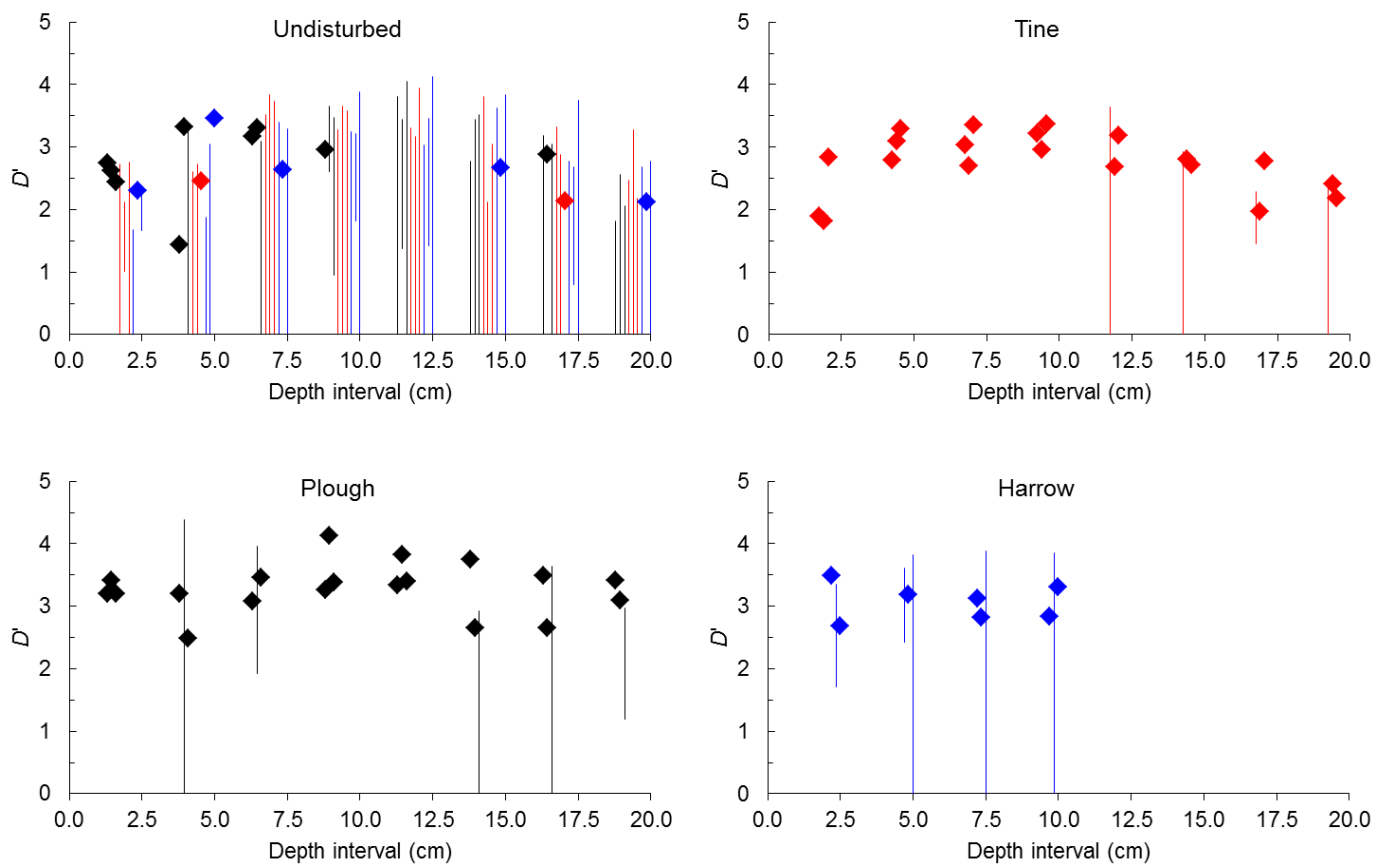
Value (points) or range (bars) of size-number distributions of seeds expressed by *D*’ for all seeds (spherical plus non-spherical) before tillage (undisturbed) and after tine, harrow or plough at 2.5 cm depth intervals.

Starting with all seeds (Figure 2), before tillage, in undisturbed soil self-similarity was only found in 22% of samples (33% considering only the top 10 cm). When present in undisturbed soil self-similarity occurred predominantly in the top 10 cm (75% of samples). After tillage by tine, self-similarity rose from 22% (8% of samples in the plot assigned to tine) to 83%. After tillage by harrow, self-similarity rose from 33% (25% of samples in the plot assigned to harrow) to 58%. After tillage by plough self-similarity rose from 22% (38% of samples in the plot assigned to plough) to 79%.

In black, samples from the plot assigned to plough; in red, from the plot assigned to tine; in blue, from the plot assigned to harrow. Points indicate self-similarity, bars indicate self-affinity.

As happened when *D*’ was analyzed for seeds from all depth intervals pooled together, when self-similarity was present before tillage it predominated in samples from the plot that was latter to be ploughed while the plot that was randomly assigned to tine showed the lesser frequency of self-similar samples.

**Figure 3:**
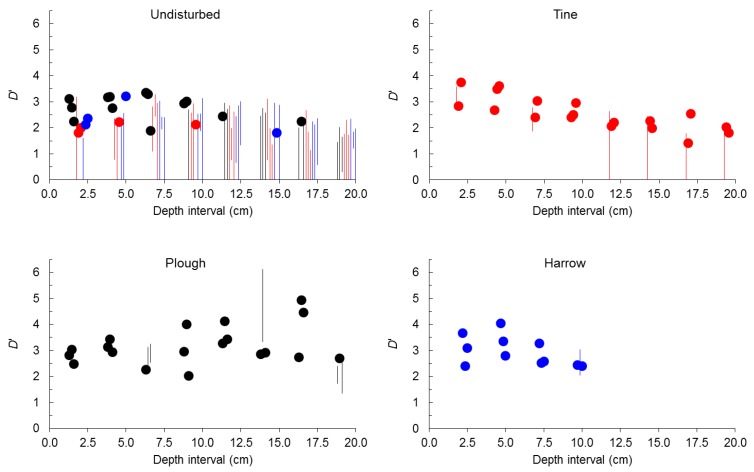
Value (points) or range (bars) of size-number distributions of seeds expressed by *D*’ for spherical seeds before tillage (undisturbed) and after tine, harrow or plough at 2.5 cm depth intervals.

Considering spherical seeds alone (Figure 3), before tillage, in undisturbed soil, self-similarity was only found in 29% of samples (50% considering only the top 10 cm). Self-similarity when present in undisturbed soil occurred predominantly in the top 10 cm (86% of samples). After tillage by tine, self-similarity rose from 29% (17% of samples in the plot assigned to tine) to 75%. After tillage by harrow, self-similarity rose from 50% (25% of samples in the plot assigned to harrow) to 92%. After tillage by plough self-similarity rose from 29% (54% of samples in the plot assigned to plough) to 79%.

In black, samples from the plot assigned to plough; in red, from the plot assigned to tine; in blue, from the plot assigned to harrow. Points indicate self-similarity, bars indicate self-affinity.

As happened when *D*’ was analyzed for seeds from all depth intervals pooled together and for all seeds (spherical plus non-spherical) separately for the eight depth intervals, when self-similarity was present before tillage it predominated in samples from the plot that was latter to be ploughed, but contrary to those analyses the plot that was randomly assigned to tine had the same frequency of self-similar samples than the plot assigned to harrow.

**Figure 4:**
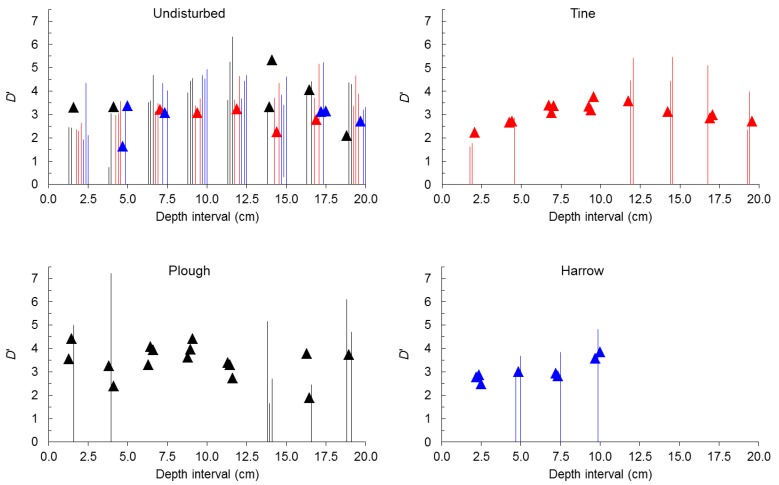
Value (points) or range (bars) of size-number distributions of seeds expressed by *D*’ for non-spherical seeds before tillage (undisturbed) and after tine, harrow or plough at 2.5 cm depth intervals.

Considering non-spherical seeds alone (Figure 4), before tillage, in undisturbed soil self-similarity was only found in 24% of samples (19% considering only the top 10 cm). Contrary to all seeds and spherical seeds, when self-similarity was present in undisturbed soil it occurred predominantly in the bottom 10 cm (59% of samples). After tillage by tine, self-similarity rose from 24% (21% of samples in the plot assigned to tine) to 58%. After tillage by harrow, self-similarity rose from 19% (8% of samples in the plot assigned to harrow) to 67%. After tillage by plough self-similarity rose from 24% (25% of samples in the plot assigned to plough) to 67%.

Contrary to what happened when *D*’ was analyzed for seeds from all depth intervals pooled together, for all seeds (spherical plus non-spherical), and for spherical seeds alone separately for the eight depth intervals, when self-similarity was present before tillage in non-spherical seeds it was evenly distributed among plots.

In black, samples from the plot assigned to plough; in red, from the plot assigned to tine; in blue, from the plot assigned to harrow. Points indicate self-similarity, bars indicate self-affinity.

Clearly self-affinity prevailed in undisturbed soil and shifted to self-similarity after tillage either when all seeds (spherical plus non-spherical), spherical seeds or non-spherical seeds were examined. In general, before or after tillage, the frequency of self-similarity was higher in spherical seeds. In undisturbed soil, before tillage, self-similarity was always more frequent in the plot that was later ploughed followed by the plot that was later harrowed in all seeds and spherical seeds, but not in non-spherical seeds where the plot that was later tined was second. Given the setup of plots in the field, as plough was located higher, harrow lower and tine intermediate (see Figure 6b,h, below in Experimental Section) differences in the frequency of self-similarity can hardly be attributed to slope.

**Figure 5:**
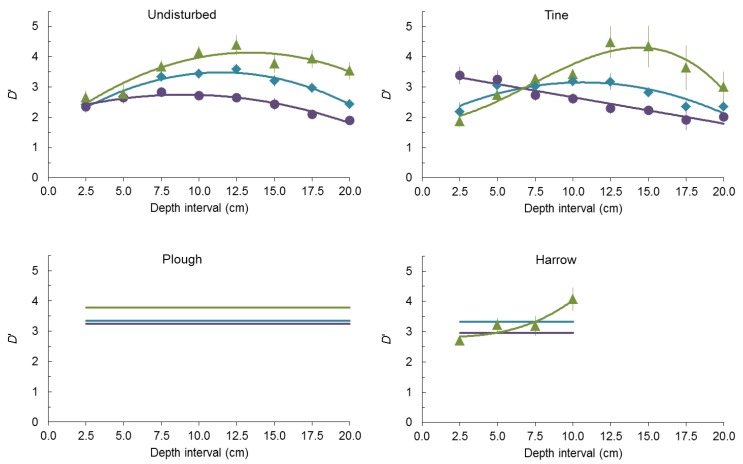
Relationship between *D*’ or the larger *D*’ (mean ± SE) and 2.5 cm depth intervals before tillage (undisturbed) and after tine, harrow or plough.

Conversely, soil depth plays a role in the change of the larger *D*’ value in undisturbed soil and in tilled soil, especially by tine (Figure 5). Fitting a polynomial equation to describe the relationship between the larger *D*’ value and soil depth was always possible in undisturbed soil, either in all seeds (*p* ≤ 10^−4^ for coefficients, lack of fit with *p* = 0.662, *R*^2^ = 0.947), in spherical seeds (*p* ≤ 0.001 for coefficients, lack of fit with *p* = 0.916, *R*^2^ = 0.957) and in non-spherical seeds (*p* ≤ 10^−4^ for coefficients, lack of fit with *p* = 0.423, *R*^2^ = 0.857).

Blue and diamonds all seeds (spherical plus non-spherical); purple and circles spherical seeds; green and triangles non-spherical seeds. Equations for undisturbed soil, all seeds *D*’ = 1.592 + 0.330*d* − 0.014*d*^2^; spherical seeds *D*’ = 2.113 + 0.140*d* − 0.008*d*^2^; non-spherical seeds *D*’ = 1.595 + 0.381*d* − 0.014*d*^2^. Equations for tillage by tine, all seeds *D*’ =1.836 + 0.248*d* − 0.012*d*^2^; spherical seeds *D*’ = 3.533 − 0.087*d*; non-spherical seeds *D*’ = 1.856 + 0.035*d*^2^ − 0.002*d*^3^. Equation for tillage by harrow, non-spherical seeds *D*’ = 2.818 + 0.001*d*^3^.

In all cases the larger *D*’ value first increased and then decreased with depth but at different rates. In all seeds the larger *D*’ increased down to 10–12.5 cm depth interval, in spherical seeds down to 5–7.5 cm depth interval and in non-spherical seeds again down to 10–12.5 cm interval. Overall, in undisturbed soil higher *D*’ values were found in non-spherical seeds, lower in spherical seeds with all seeds intermediate.

Fitting a polynomial equation to describe the relationship between the larger *D*’ value and soil depth was always possible after tine either in all seeds (*p* ≤ 4 × 10^−4^ for coefficients, lack of fit with *p* = 0.333, *R*^2^ = 0.792), in spherical seeds (*p* ≤ 10^−4^ for coefficients, lack of fit with *p* = 0.847, *R*^2^ = 0.938) and in non-spherical seeds (*p* ≤ 10^−4^ for coefficients, lack of fit with *p* = 0.859, *R*^2^ = 0.925). In all seeds and non-spherical seeds the larger *D*’ value first increased and then decreased with depth but at different rates. In all seeds the larger *D*’ increased down to 7.5–10 cm depth interval and in non-spherical seeds down to 10–12.5 cm depth interval while in spherical seeds the larger *D*’ value decreases monotonically with depth. Overall, after tine higher *D*’ values were found in spherical seeds down to 5 cm depth, in non-spherical seeds at deeper depths with all seeds always intermediate.

**Figure 6:**
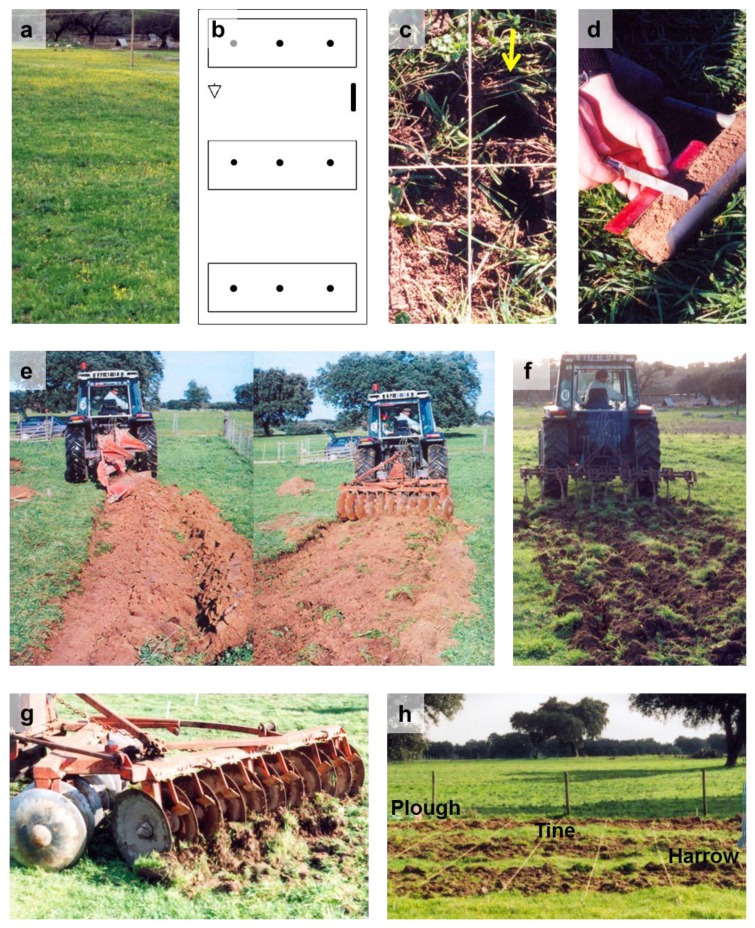
(**a**) General view of area of study; (**b**) Experimental scheme showing plots (top down, plough, tine, harrow), location of sampled cores, direction of slope (arrow), with black vertical bar representing 1 m; (**c**) Orthogonal grid with sample location (arrow); (**d**) Soil core measurement; (**e**) Plough; (**f**) Tine; (**g**) Harrow; (**h**) General view with location of plots after tillage.

Fitting a polynomial equation to describe the relationship between the larger *D*’ value and soil depth after harrow was only possible for non-spherical seeds (*p* ≤ 0.006 for coefficients, lack of fit with *p* = 0.558, *R*^2^ = 0.896) with the larger *D*’ value increasing monotonically with depth. Conversely, no equation could be fitted to all seeds and to spherical seeds, and no significant differences were found among depth intervals (*p* = 0.801 for all seeds, *p* = 0.360 for spherical seeds). Pooling together all values, the larger *D*’ value was 3.330 ± 0.077 in all seeds and 2.964 ± 0.168 in spherical seeds. No significant differences in the larger *D*’ value was found between all seeds and spherical seeds after harrow (*p* = 0.078). Overall, after harrow higher *D*’ values were found in all seeds down to 7.5 cm depth, in non-spherical seeds at 7.5–10 cm depth interval with spherical seeds almost always having the lower *D*’ values.

Fitting a polynomial equation to describe the relationship between the larger *D*’ value and soil depth after plough was never possible and no significant differences were found among depth intervals (*p* = 0.911 for all seeds, *p* = 0.353 for spherical seeds, *p* = 0.398 for non-spherical seeds). Pooling together all values, the larger *D*’ value was 3.347 ± 0.062 in all seeds, 3.249 ± 0.197 in spherical seeds and 3.779 ± 0.256 in non-spherical seeds. No significant differences in the larger *D*’ value was found among all seeds, spherical seeds and non-spherical seeds after plough (*p* ≈ 1).

Broadly speaking, in undisturbed soil, self-affinity was largely prevalent meaning that size-number distribution of seeds was not random but depended upon the size of seeds itself. Thus, it would depend on the functional differences among seeds of different sizes, which would respond differently to past environmental conditions and constraints. However, this adaptive response of the soil seed bank to past environmental conditions and constraints is clearly disrupted by tillage, almost irrespective of the intensity of the disturbance it imposed, with soil seed banks showing a generalized pattern of randomness of seed size distribution after either tine, harrow or plough.

Randomness of seed distribution after tillage was previously stated [12,41] and experimentally recognized [42] for plough, but not for other types of cultivation like harrow or tine, which is now done. Considering size-number distributions of seeds of soil seed banks randomness is clearly the result of all the above types of cultivation, thus suggesting that even in no-till cultivation the inevitable disturbance imposed by seeding might break self-affinity of seed-number distributions and disrupt weed ecological adaptations it represents.

However, and not surprisingly, tillage of high intensity of disturbance like plough differs from tillage of low intensity of disturbance like tine because in addition to self-affinity the relationship between *D*’ and depth is also broken and differences between spherical and non-spherical seeds disappear in plough. Conversely, the relationship between *D*’ and depth found in undisturbed soil is still recognizable after tine with minor differences at shallower depths.

Fractal dimension was found to range between 1.07 and 1.41 in landscape topography [43], was < 2 for the above-ground distribution of three weed species [44], of boreal forests [45] and of a variety of soil parameters and geographical and geophysical data [38], around 2 in a variety of other plant communities [46], between 2 and 3 in various forest types [47], ranged between 2.68 and 3.49 for soil particle-size distribution [48,49], and sometimes higher than 5 [49,50].

Considering only samples after tillage, *D*’ ranged between 1.41 and 4.93 in self-similar samples with a mean value of 3.05 ± 0.05 and between 1.41 and 7.23 with a mean value of 3.22 ± 0.06 when the larger *D*’ value of self-affine samples is also included. These values clearly put soil seed banks of soils disturbed by tillage in the higher end of *D*’ spectrum presented above. Thus, according to [38], factors governing seed-size distributions of seeds immediately after tillage should have short-range, short-term variation which is not surprising and was to be expected given not only the intensity of disturbance impose by tillage but also the very small time that elapsed between the first sampling, cultivation by tine, harrow or plough and seed banks sampling after tillage.

However, *D*’ was not significantly different before and after tillage. Before tillage the fractal dimension *D*’ of self-similar samples ranged between 1.43 and 5.32 (mean value 2.76 ± 0.08) and between 0.75 and 6.34 (mean value 3.07 ± 0.05) when the larger *D*’ value of self-affine samples is also included. It is true that before and after tillage *D*’ is significantly different (*p* = 0.002) when only self-similar samples are compared but this difference disappears (*p* = 0.05) when the larger value of self-affine samples is included in the analysis.

Altogether these results imply, even in the absence of short-time, short-range disturbances imposed by tillage factors governing size-number distribution of soil seed banks still operate at remarkably short-time, short-range levels. The fast response of size-number distribution of soil seed banks to environmental pressures implied by these results is even more noticeable because it results from sampling soil seed bank after the germination of the most part of constituents of transient seed bank fraction. Thus, only the short and long-term persistent fractions of the seed bank as defined and adopted in [51] are likely to be involved, those being the fractions that were subjected to longer environmental pressures.

It remains an open question whether such short-range dependency is a particular adaptation of plants thriving in the notoriously unpredictable Mediterranean environment where this study was conducted or a general feature of soil seed banks dominated by therophytes.

## 3. Experimental Section

### 3.1. Location, Soil Seed Bank Sampling and Tillage Experiment

Field work was done in Herdade Experimental da Mitra (Mitra Experimental Farm), Universidade de Évora, located near Évora, Southern Portugal (38° 32' N, 8° 1' W). The site was an area of open *montado* of holm oak (*Quercus ilex* L.) with natural pasture and a gentle slope (Figure 6a). Cultivation or cropping has not been done in the experimental site for more than 10 years and sheep grazing occurred very rarely and with low intensity. Soil was sandy loam with 50% coarse sand, 24% fine sand, 12% silt, 14% clay (analyses done by Laboratório Químico Agrícola, Universidade de Évora).

Three plots perpendicular to the slope, 6 × 2 m^2^ each and 3 m apart were defined and each plot randomly assigned to one tillage treatment (Figure 6b). By mid-winter, before tillage, small poles were driven into the soil well outside the plots and ropes tied to them so that the resulting orthogonal grid could be used to locate exactly sample places before and after tillage (Figure 6c). Then three soil cores 5 cm Ø and 20 cm depth were taken in each plot with an auger at 2 m intervals in a straight line. Each soil core was divided with a knife in eight 2.5 cm fractions (Figure 6d), each portion separately stored at −30 °C until being processed.

Four days after the first sampling, ropes were removed and tillage was done following the best agronomic practices. Plough was done with a two-furrow moldboard (three passes, reversing direction at each pass) followed by one pass of disk harrow with eleven 24''-blades (Figure 6e). Depth of tillage ranged between 18 and 22 cm. Tine cultivation was done with a spring tine cultivator (four passes, reversing direction at each pass) at a depth of approximately 20 cm (Figure 6f). Harrowing was done with a disk harrow with eleven 24''-blades (five passes, reversing direction at each pass) but due to soil compaction the operation did not go below 8–10 cm depth (Figure 6g).

Five days after tillage ropes were again tied to poles left in the field (Figure 6h) and sampling was repeated in the same places, except that harrow sampling was only done down to 10 cm-depth.

### 3.2. Sample Processing

Samples were taken from the freezer as needed and kept two days at room conditions before being processed. Each sample (a cylinder 5 cm Ø, 2.5 cm height) was sequentially sieved with hand disaggregation under a gentle stream of hot water through a series of ten sieves 2.38, 0.85, 0.71, 0.56, 0.425, 0.355, 0.297, 0.25, 0.212 and 0.149 mm mesh side. Fractions retained by sieves with mesh side 0.355 mm or higher, composed by coarser materials and clearly visible organic matter were separately transferred to Whatman 540 paper, excess water removed by suction, materials dried in an electric oven at 60 °C and stored before seeds were sorted and counted. Fractions retained by sieves with mesh side 0.297 mm or smaller were sunk in 25 mL of magnesium sulfate distilled water solution (250 g L^−1^), gently stirred during two minutes in order to separate the mineral component from the organic component seeds included and after two additional minutes of rest, floating materials were transferred to Whatman 540 paper [52,53], excess water removed by suction, materials dried in an electric oven at 60 °C and stored before seeds were sorted and counted.

Fractions retained by sieves 0.85 mm or lower were examined under a stereomicroscope while those retained by 2.380 mm mesh side were examined with naked eye. Seeds were considered viable according to their resistance to pressure by tweezers [54], classified either as spherical or roughly spherical or as clearly non-spherical, and counted according to their shape. Due to the very high number of seeds, shape classification had to be done visually.

Ten random samples of the mineral fraction of the 0.297 mm or lesser meshes and of the materials not retained by the 0.149 mm mesh were processed and inspected for lost seeds as described above.

No attempt was done to identify the species of each and every seed but only the identification of all species present in all samples. Identification was done using published seed identification guides [55,56,57,58,59] and seeds photographs taken by the author. Species nomenclature follows The Plant List [60].

### 3.3. Modeling Seed-Size Distributions

The approach and procedures of Casco *et al*. [30] were generally followed including the choice of mesh side instead of mesh bisector as a surrogate for seed size. All statistics were done with Statgraphics 4.2 (STSC, Inc., Rockville, MD, USA) except Box-Cox transformations done with BIOM (Applied Biostatistics, Inc., New York, NY, USA) and lack of fit tests done with Excel^®^2010 (Microsoft Corporation).

#### 3.3.1. Power Law

The power law is expressed as:

*P_S_*_>*s*_ = *F s^D^* (1)

where *P_S_*_>*s*_ is the proportion of seeds greater than a given size which is equated with the mesh side *s* that retained them; *F* and *D* (*D* < 0) are constants. *F* is the value of *P_S_*_>*s*_ when *s* = 1 and *D* is equivalent to the Haussdorff-Besicovich dimension. Using logarithms Equation (1) is rendered linear in the form:

ln *P_S_*_>*s*_ = ln *F* + *D* ln *s* (2)

Expressing the mesh side *s* in proportion to the smaller mesh side *s*_min_ which is known in any given sample (almost always *s*_min_ = 0.149 mm), then *s’* = *s*/*s*_min_ and Equation (2) reduces to:

ln *P_S_*_>*s*’_ = *D* ln *s*’ (3)

because when *s* = *s*_min_ the proportion *P*_Y>*s*’_ of seeds greater than the mesh side *s*_min_ is necessarily unity, implying that ln *F* = 0.

Equation (3) describes a self-similar power model of seed-size distribution in which the relationship between seed size expressed by ln *s*’ and seed distribution expressed by ln *P_S_*_>*s*’_ is constant across the whole range of *s*’. However, the relationship between ln *s*’ and ln *P_S_*_>*s*’_ may not be constant across all values of *s*’ requiring additional terms in Equation (3). Thus Equation (3) can be seen as a particular case of a more general relationship between ln *s*’ and ln *P_S_*_>*s*’_ that can be expressed by:

ln *P_S_*_>*s*’_ = *D* ln *s*’ + *A* (ln *s*’)^2^ + *B* (ln *s*’)^3^ + *C* (ln *s*’)^4^ (4)

that describes a self-affinity power model of seed-size distribution that reduces to the self-similar model when *A* = *B* = *C* = 0.

The reparameterized power function presented in Equation (4) was fitted by stepwise regression without replication forced through the origin using the least squares method with an experiment-wise confidence level for coefficients of *p* = 0.05 calculated by the Dunn-Šidák method [61]. Whenever samples had size number *n* ≤ 4 stepwise regression was replaced by fitting separately all possible one-term models, adding terms in all possible combinations and testing the increase of the coefficient of determination *R*^2^ using the *F* distribution and a significance level of *p* = 0.05 [62].

Equations only accepted after checking that ln *P_S_*_>*s*’_ ≤ 0 for any value of ln *s*’ and that ln *P_S_*_>*s*’_ decreased monotonically with ln *s*’. Whenever equations failed to comply with one of these conditions, equations were fitted again either by removing the term with the higher significance level or by adding separately all terms absent from the equation and testing the increase of the coefficient of determination *R*^2^ using the *F* distribution and a significance level of *p* = 0.05 [62].

After being fitted and accepted, equations were back-transformed as:

*P_S_*_>*s*’_ = *s*’ ^*D* + *A* ln *s*’ + *B* (ln *s*’)^2^ + *C* (ln *s*’)^3^^ (5)

and for each equation the smaller and larger value of

*D*’ = − [*D* + *A* ln *s*’ + *B* (ln *s*’)^2^ + *C* (ln *s*’)^3^] (6)

was determined for the whole range of *s*’ values and the resulting values of the fractal dimension *D*’ used as a measure of self-similarity (*D*’ = −*D* and constant in the whole range of *s*’ values) or self-affinity (*D*’ ≠ −*D* and variable with *s*’) of seed-size distribution.

#### 3.3.2. Statistical Analyses

Comparisons of means involving only two samples were made by exact two-tailed Student’s *t* tests after checking for homocedasticity with the two-tailed *F* distribution. Comparisons of means involving more than two samples were made by single classification ANOVA after checking for homocedasticity with the two-tailed *F* distribution. Whenever heterocedasticity was found for *p* = 0.05 data was transformed prior to ANOVA using the Box-Cox transformation [63]. The relationship between soil depth and seed-size distribution described by the larger *D*’ calculated from Equation (6) was investigated fitting up to third order polynomials by stepwise regression with replication using the least squares method and an experiment-wise confidence level for coefficients of *p* = 0.05 calculated by the Dunn-Šidák method [61]. Coefficients of determination (*R*^2^) are presented as proportion of the maximum *R*^2^ possible [64].

## 4. Conclusions

Power law and the resulting analogs of fractal and multifractal dimensions can be used to characterize size-number distributions of soil seed banks and the effects of soil disturbances on them.

In the absence of soil disturbance by tillage soil seed bank responses to past events results in the prevalence of self-affinity, meaning that size-number distributions are not independent from seed size itself.

Soil disturbance by tine, harrow or plough breaks this dependency and is immediately reflected in the shift from self-affinity to self-similarity of size-number distribution of soil seed banks, meaning that tillage imposes randomness to size-number distribution regardless of the intensity of soil disturbance induced by tillage.

As could be expected, the magnitude of fractal dimensions after tillage shows that size-number distributions of soil seed banks responded to short-term, short-range factors. However, before and after tillage the values of fractal dimensions were almost the same, which means that in undisturbed soils the size-number distributions of soil seed banks were also being affected and responding to short-term, short-range factors.
